# Do Organic Amendments Foster Only Beneficial Bacteria in Agroecosystems?: The Case of *Bacillus paranthracis* TSO55

**DOI:** 10.3390/plants14071019

**Published:** 2025-03-25

**Authors:** Ixchel Campos-Avelar, Amelia C. Montoya-Martínez, Alina Escalante-Beltrán, Fannie I. Parra-Cota, Sergio de los Santos Villalobos

**Affiliations:** 1 Laboratorio de Biotecnología del Recurso Microbiano, Departamento de Ciencias Agronómicas y Veterinarias, Instituto Tecnológico de Sonora, 5 de Febrero 818, Col. Centro, Cd. Obregón 85000, Mexico; ixchel_campos_avelar@hotmail.com (I.C.-A.); cristina_montoya14@hotmail.com (A.C.M.-M.);; 2 Campo Experimental Norman E. Borlaug—Instituto Nacional De Investigaciones Forestales, Agrícolas y Pecuarias (INIFAP), Norman E. Borlaug Km. 12, Cd. Obregón 85000, Mexico; parra.fannie@inifap.gob.mx

**Keywords:** organic amendments, pathogenic strains, bioinoculants, taxonomic affiliation, genome mining

## Abstract

Bacterial strain TSO55 was isolated from a commercial field of wheat (*Triticum turgidum* L. subsp. *durum*), under organic amendments, located in the Yaqui Valley, Mexico. Morphological and microscopical characterization showed off-white irregular colonies and Gram-positive bacillus, respectively. The draft genome sequence of this strain revealed a genomic size of 5,489,151 bp, with a G + C content of 35.21%, N50 value of 245,934 bp, L50 value of 8, and 85 contigs. Taxonomic affiliation showed that strain TSO55 belongs to *Bacillus paranthracis*, reported as an emergent human pathogen. Genome annotation identified 5743 and 5587 coding DNA sequences (CDSs), respectively, highlighting genes associated with indole production, phosphate and potassium solubilization, and iron acquisition. Further in silico analysis indicated the presence of three CDSs related to pathogenicity islands and a high pathogenic potential (77%), as well as the presence of multiple gene clusters related to antibiotic resistance. The in vitro evaluation of plant growth promotion traits was negative for indole production and phosphate and potassium solubilization, and it was positive but low (18%) for siderophore production. The biosynthetic gene cluster for bacillibactin (siderophore) biosynthesis was confirmed. Antifungal bioactivity of strain TSO55 evaluated against wheat pathogenic fungi (*Alternaria alternata* TF17, *Bipolaris sorokiniana* TPQ3, and *Fusarium incarnatum* TF14) showed minimal fungal inhibition. An antibiotic susceptibility assay indicated resistance to three of the six antibiotics evaluated, up to a concentration of 20 µg/mL. The beta hemolysis result on blood agar reinforced TSO55’s pathogenic potential. Inoculation of *B. paranthracis* TSO55 on wheat seedlings resulted in a significant decrease in root length (−8.4%), total plant height (−4.2%), root dry weight (−18.6%), stem dry weight (−11.1%), and total plant dry weight (−15.2%) compared to the control (uninoculated) treatment. This work highlights the importance of analyzing the microbiological safety of organic amendments before application. Comprehensive genome-based taxonomic affiliation and bioprospecting of microbial species introduced to the soil by organic agricultural practices and any microbial inoculant will prevent the introduction of dangerous species with non-beneficial traits for crops, which affect sustainability and generate potential health risks for plants and humans.

## 1. Introduction

Intensive agricultural practices employed for food production have allowed farmers to increase crop yields during the last decades. However, they have also caused environmental pollution, soil erosion, physicochemical degradation, and the loss of microbial diversity [1]. For this reason, over many years, diverse strategies have been implemented to promote agricultural soils’ enrichment, by focusing mainly on increasing their physical, chemical, and biological fertility. In this sense, the addition of organic amendments (i.e., manure, food residues, compost, etc.) to the soil to revive microbial activity is a widely implemented approach to improve soil properties [2]. Thus, organic amendments represent an effective strategy for increasing the amount of organic matter and microbial diversity in nutrient-depleted soil [3].

However, due to their provenance (derived from plant and animal residues in decomposition), in some cases, the addition of organic amendments can introduce pathogenic strains to the soil and/or increase their relative abundance, which can represent a potential risk for crops, animals, and humans, especially for farmers who work in direct contact with airborne dust and droplets [4,5]. Thus, it is essential to correctly identify the types of microorganisms introduced to the agricultural ecosystem through the application of organic amendments, to explore their ecological and environmental role, and to ensure their safe and proper deployment, as well as to guarantee food safety and sustainability [6].

The lack of these safety precautions could cause unintended adverse impacts on human health, through the introduction of pathogenic strains in agricultural soils or by favoring their development. Furthermore, these practices have been linked to the increase in antimicrobial resistance (AMR) of pathogenic strains in agricultural soils, as they represent a major reservoir of antibiotic residues [7,8]; in addition, antimicrobial resistance and virulence factor genes can be transmitted to native bacteria through horizontal gene transfer [5]. Moreover, land application of organic amendments based on sludge or animal manure has been associated with the dispersion of pathogenic microorganisms through water runoff and bioaerosols [9]. For instance, pathogens such as *Clostridium botulinum*, *Listeria monocytogenes*, *Salmonella enterica*, and *Escherichia coli* can be present in vegetables’ and fruits’ agricultural production systems, due to irrigation with contaminated wastewater and/or the use of animal manure as fertilizers [10].

Regarding wheat crops, although grain and flour contamination with pathogenic bacteria is less common—due to their low water activity, and the fact that wheat products are not intended to be consumed raw—recalls and related illness outbreaks have been increasingly associated with wheat flours and their derivatives. This could be attributed to an increase in wheat-based foods, improper grain storage practices, and unsanitized farming equipment [11,12]. Furthermore, even though grain processing and manufacturing reduces the risk of microbial contamination, some microbial species, such as *Salmonella* and *Escherichia coli*, as well as their toxins, are capable of remaining on wheat flour or wheat-based products [11,12]. Moreover, post-harvest contamination and milling of contaminated grains have been found to facilitate microbial distribution to wheat flour and other resulting fractions [11]. Due to the potential risk, mitigation approaches for the safe use of organic amendments are highly pertinent, which include correct stabilization/disinfection processes, restricted application to crops that require cooking before consumption (e.g., potatoes), and injection rather than dispersal of organic amendments, among others [9].

According to the literature, several microbial strains are reported as plant growth-promoting and biological control agents, despite possessing pathogenic traits [13,14,15], such as *Enterobacter cloacae* [16], *Pseudomonas putida* [17], *Bacillus thuringiensis* [18], *B. cereus* [19], and *Burkholderia cepacia* [20]. On the other hand, *Bacillus* is largely considered a beneficial agent, and its presence on the field is seen as something generally positive [21,22,23]. However, it is important to highlight that, although this genus can promote plant growth and/or inhibit phytopathogenic species, the presence of some species in the agroecosystem could negatively impact the health of animals (humans included) consuming food originating from those areas [7], as some *Bacillus* species have been reported as possessing pathogenic traits [23]. Thus, it is crucial to perform a correct taxonomic affiliation of the soil microbiome to identify which specific species are present and the potential benefits or risks they represent [24].

At present, a major advance in agricultural microbiology is the use of bioinformatic tools for accelerating both the taxonomic affiliation of microbial strains and their bioprospection [25]. Through the use of genomics, it is possible to analyze the curated DNA information of a microorganism and to compare it with global databases to determine if the species is already reported or if it is a new taxon [26]. In both cases, the available information in the literature and bioinformatic indices allows us to predict the putative benefits and/or risks of the studied organism.

Furthermore, the exploration of genes, enzymes, and metabolites involved in plant growth promotion, pathogenic biocontrol, and other potential biological functions is possible thanks to the implementation of genome mining, and their confirmation can be determined by using in vitro and in vivo assays [25,27,28,29]. Thus, these cutting-edge technologies considerably reduce the time invested in identifying and screening microorganisms of interest; however, it is essential to experimentally confirm the predicted biological applications.

Hence, this study aimed to perform morphological and functional characterization, as well as genome-based taxonomic affiliation and genome mining, of a bacteria (strain TSO55) isolated from agricultural soil used for wheat production with the application of organic amendments for 10 years. To achieve this, diverse predicted traits were evaluated in vitro, such as plant growth promotion, biocontrol capacity, pathogenic traits, and antibiotic susceptibility.

## 2. Results

### 2.1. Morphology of Strain TSO55

**Figure 1:**
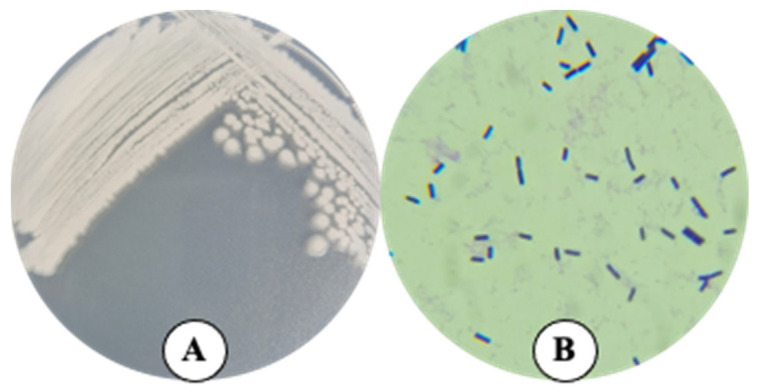
Growth of strain TSO55 on nutrient agar medium (**A**). Microscopical observation (100×) of bacterial cells of strain TSO55 showing a rod-shaped form and positive Gram staining (**B**).

After being recovered from frozen glycerol stock and cultured on Petri dishes containing nutrient agar (NA) as a culture medium, at 28 °C for 24 h, strain TSO55 showed round, off-white, opaque colonies with slightly lighter irregular borders. Under the microscope, strain TSO55 showed bacterial cells with a non-grouped rod-shaped form and Gram-positive staining (Figure 1).

### 2.2. Genomic Analysis

**Figure 2:**
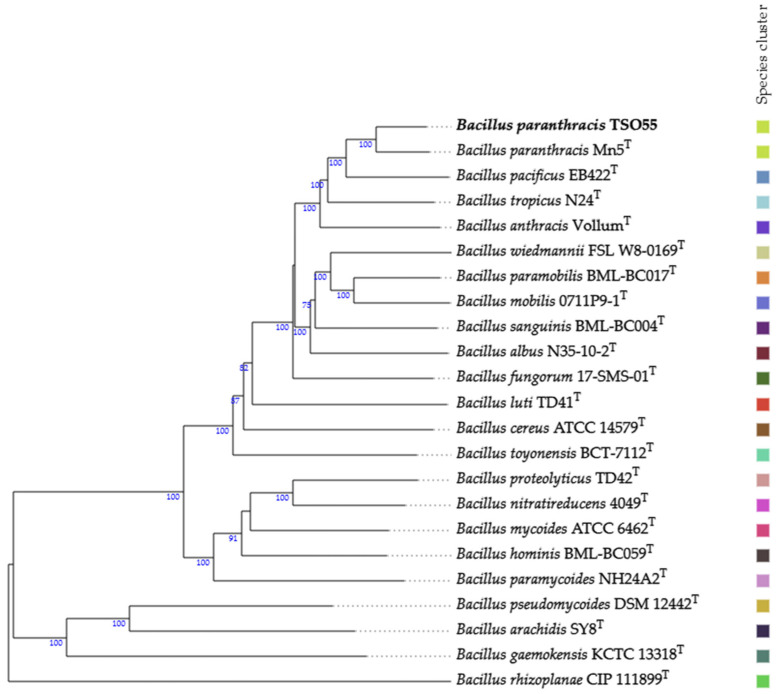
Phylogenomic relationship between strain TSO55 and closely related species based on genome sequences constructed by TYGS. Tree inferred with FastME 2.1.6.1 [30] from GBDP distances calculated from genome sequences. The branch lengths are scaled in terms of the GBDP distance formula d5. The numbers above the branches are GBDP pseudo-bootstrap support values > 60% from 100 replications, with an average branch support of 73.0%. The tree was rooted at the midpoint [31]. The same color in the color scale on the left indicates the same species, according to the calculated phylogenetic relationship. ^T^ = Type strain.

The genome sequencing of strain TSO55 yielded a total of 884,192 paired-end reads (2 × 250 bp). The subsequent trimming and assembly of these reads resulted in the draft genome of 85 contigs (≥200 bp). The assembled genome had a total length of 5,489,151 bp, with a G + C content of 35.21%, an N50 value of 245,934 bp, and an L50 of 8. Notably, no plasmids or contamination was detected within the genome of strain TSO55. According to the 16S rRNA gene, strain TSO55 showed 100% similarity to *Bacillus paranthracis* Mn5 ^T^, 100% to *B. nitratireducens* 4049 ^T^, 100% to *B. tropicus* N24 ^T^, 100% to *B. anthracis* Ames, and 99.92% to *B. cereus* ATCC 14579 ^T^ (Table 1). Based on the OGRI analysis, this strain was strongly affiliated with *Bacillus paranthracis*, due to these values being higher than those delimiting the species affiliation (ANI ≥ 95–96% and GGDC ≥ 70%) (Table 1). Finally, this taxonomic affiliation was confirmed by the construction of a whole-genome-based phylogenomic tree (Figure 2), showing the close evolutionary relationship of strain TSO55. Thus, according to the obtained results, strain TSO55 belongs to *Bacillus paranthracis*.

### 2.3. Genome Annotation and Genome Mining

**Figure 3:**
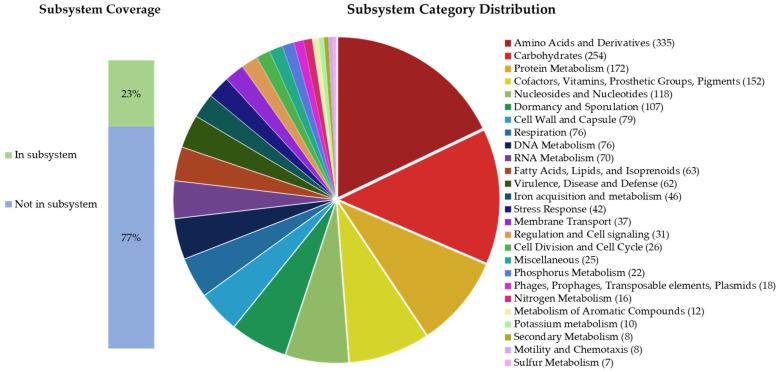
Pie chart of the subsystem category distribution of CDSs from *Bacillus paranthracis* TSO55.

According to the RAST prediction, the genome of *B. paranthracis* TSO55 contains 117 RNA sequences and 5743 protein-coding DNA sequences (CDSs), of which 23% are distributed across 336 subsystems, as shown in Figure 3.

Notable subsystems in the genome of strain TSO55 include coding DNA sequences associated with potential plant growth promotion, such as (i) iron acquisition and metabolism (46 CDSs) [siderophores (24 CDSs)], and (ii) secondary metabolism (8 CDSs) [auxin biosynthesis (4 CDSs)]. In addition, subsystems related to stress response (42 CDSs) were detected, including genes for the responses to osmotic (11 CDSs) and oxidative stress (18 CDSs), as well as to detoxification (2 CDSs) (Figure 3). Moreover, when annotating the genome of TSO55 with RAST, in the subsystem phages, prophages, transposable elements, and plasmids (18 CDSs), 3 CDSs of pathogenicity islands were found: listeria pathogenicity island, two phospholipase C and one thiol-activated cytolysin. On the other hand, RAST also predicted the subsystem of virulence, disease, and defense (62 CDSs), where the subcategory resistance to antibiotics and toxic compounds (47 CDSs) showed genes for beta-lactamase (1 CDS) and multidrug (6 CDSs), tetracycline (4 CDSs), streptothricin (1 CDS), fosfomycin (1 CDS), and fluoroquinolones (2 CDSs) resistance, as well as resistance to copper (7 CDSs), chromium (1 CDS), and cobalt–zinc–cadmium (20 CDSs). Additionally, the AntiSMASH 7.1 web server was used for mining the genome of *Bacillus paranthracis* TSO55, resulting in one biosynthetic gene cluster, bacillibactin (NRP-metallophore), with a similarity of 85%.

**Figure 4:**
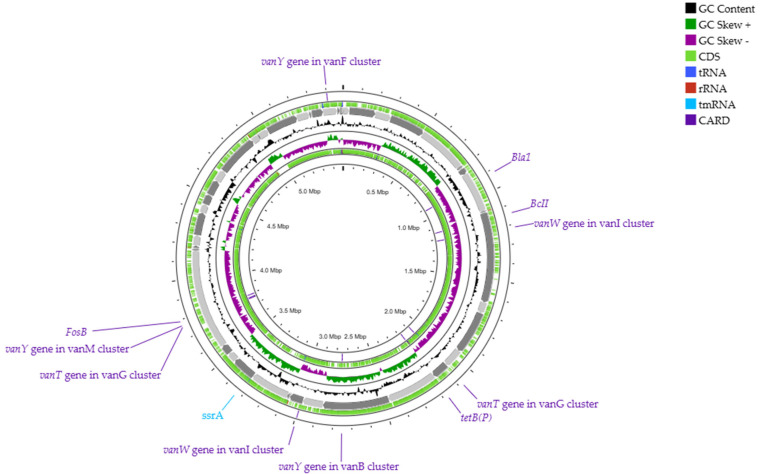
Circular chromosome map of *Bacillus paranthracis* TSO55, including the distribution of CDSs, tRNAs, rRNAs, and GC content skew generated through Prokka genome annotation. Additionally, the identified antibiotic resistance genes found by the CARD Resistance Gene Identifier tool are also shown [beta-lactams (*Bla1*, *BcII*), vancomycin (*vanY*, *vanW*, *vanT*), tetracycline [*tetB(P)*], and fosfomycin (*FosB*).

Complementing these results, the circular chromosome map based on Prokka predicted a total of 5587 CDSs, 100 tRNAs, 16 rRNAs, and 1 tmRNA. Furthermore, the CARD Resistance Gene Identifier tool found eleven features including genes related to beta-lactams (*Bla1*, *BcII*), resistance to vancomycin (*vanY*, *vanW*, *vanT*) and tetracycline [*tetB(P)*], and the inactivation of the antibiotic fosfomycin (*FosB*) (Figure 4), which concur with the main CDSs identified by RAST. The pathogenicity of *B. paranthracis* TSO55 was also predicted in silico, using the online tool PathogenFinder. The genome sequence of this strain matched a total of 52 pathogenic families and 7 non-pathogenic families from microorganisms including *Bacillus cereus*, *B. anthracis*, and *B. thuringiensis*. Some putative protein functions found include DNA segregation, esterase, superoxide dismutase, sporulation, and transcriptomic repair. Overall, PathogenFinder results grant strain *Bacillus paranthracis* TSO55 a high pathogenic potential (77%).

**Figure 5:**
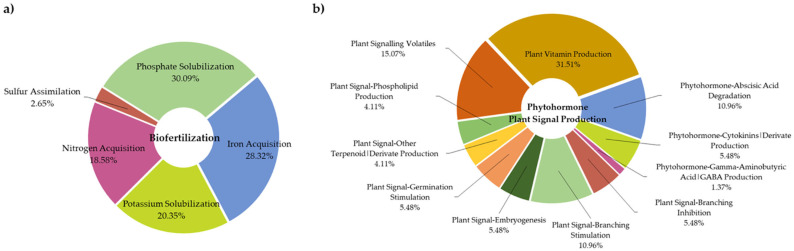
Genomic features based on PGPT-Pred analysis show (**a**) biofertilization features and (**b**) phytohormone and plant signal features.

Additionally, the PGPT-Pred predictor of PlaBase revealed the genomic features of *B. paranthracis* TSO55, which could be related to plant growth promotion. Of all of these features (884), 24.77% corresponded to colonizing plant systems, 23.53% to competitive exclusion, 19.34% to control biotic and abiotic stresses, 12.78% to biofertilization, 8.26% to phytohormone production and plant signaling, 10.63% to bioremediation, and 0.57% to plant immune response stimulation. Of note, among the features related to biofertilization (113) and phytohormone production (73), the results indicated the presence of genes for phosphate and potassium solubilization, iron and nitrogen acquisition, sulfur assimilation, plant signaling, and phytohormone production (Figure 5).

### 2.4. In Vitro Evaluation of Predicted Traits of Strain TSO55: Plant Growth Promotion, Biocontrol, Antibiotic Susceptibility, and Pathogenicity

To confirm the phenotypic expression of the obtained genomic mining results, the in vitro evaluation of plant growth promotion traits of strain TSO55 was carried out. The results indicate that strain TSO55 showed the ability to produce siderophores at a low level (18%), as a clear halo appeared around the bacterial colony grown on CAS medium. On the contrary, negative results were observed for phosphate and potassium solubilization, as the absence of a clear halo around the colony was reported. Furthermore, indole production was also negative, according to the used standard. These findings suggest that strain TSO55 does not have the most studied beneficial traits related to plant growth and development under the tested conditions.

Moreover, strain TSO55 was evaluated for its capacity to control three common fungal phytopathogens: *Alternaria alternata* TF17, *Bipolaris sorokiniana* TPQ3, and *Fusarium incarnatum* TF14, which cause diverse diseases on cereal crops. According to the obtained results, fungal growth inhibition was minimal, with 8%, 5%, and 14% of fungal growth inhibition, respectively, thus leading us to conclude that *Bacillus paranthracis* TSO55 does not possess significant antifungal capacity against the evaluated fungi, under the studied conditions.

In addition, as genome annotation of strain TSO55 indicated the presence of diverse genes for antibiotic resistance, the bacterial susceptibility to six commercial antibiotics (or antibiotic combinations) was evaluated both on NA plates by the well diffusion method and in a liquid nutrient broth medium. The results indicated that strain TSO55 was susceptible to three (ciprofloxacin, erythromycin, and clarithromycin) of the six evaluated antibiotics, with a tested minimal inhibitory concentration (MIC) of 2 µg/mL. Furthermore, the diameters of halos of inhibition increased by more than twice when a concentration of 20 µg/mL was used (Table 2). Finally, during the hemolysis assay, the results indicated that strain TSO55 possesses beta-hemolytic capacity, as a clear halo formed around the bacterial colonies grown on blood agar, which reinforces its pathogenic potential.

### 2.5. In Vivo Impact of Strain TSO55 on Wheat Growth

The inoculation of strain TSO55 on developing wheat seedlings in seedling trays filled with commercial growing medium showed a significant (*p*-value ≤ 0.05) decrease in root length (8.4%) and root dry weight (18.6%), as well as in stem dry weight (11.1%), compared to a control without bacterial inoculation; only the stem length remained unaffected by the treatment (Table 3).

## 3. Discussion

The morphology of strain TSO55 corresponded to the typical characteristics of the genus *Bacillus*, such as round white and opaque colonies and rod-shaped cells with positive Gram staining [32,33]. The genomic analysis showed a genome size of about 5.5 Mb, a G + C content of around 35%, an N50 value of 245,934 bp, and an L50 of 8. The OGRI (ANI and GGDC) values and the whole-genome-based phylogenomic tree found during this study confirmed that strain TSO55 belongs to *Bacillus paranthracis*, which corresponds to the observed morphological traits [34]. *Bacillus paranthracis* belongs to the *Bacillus cereus* group, which is constituted of at least 21 closely related species, ranging from pathogens to probiotics, such as *B. anthracis* (anthrax producer), *B. cereus* (foodborne pathogen), and *B. thuringiensis* (biopesticide) [34]. The morphological and genetic proximity of the species in this group makes it difficult to precisely identify its isolates; this becomes problematic as, while some of them are useful in agriculture and industry, several of them can cause a series of illnesses such as gastrointestinal infections, diarrhea, and anthrax [35]. For this reason, a polyphasic taxonomical approach is essential to avoid misidentifying an organism of interest that may put at risk food safety [24]. Cutting-edge technologies involving whole-genome sequencing (WGS) and access to publicly available genomes submitted to the National Center for Biotechnology Information (NCBI), as well as the average nucleotide identity (ANI) calculation, both presented in this work, have proven to be essential for characterizing *B. cereus* isolates [35].

Regarding the prediction of genomic features of strain TSO55, the results showed the presence of diverse coding sequences related to plant growth-promoting potential, such as phytohormones, plant signaling molecules, and siderophore production, as well as phosphate and potassium solubilization. However, in vitro evaluation of several of these traits showed a lack of IAA production, as well as no phosphate or potassium solubilization. On the other hand, a positive result was obtained for siderophore production, through the formation of a small yellow halo around the bacterial colony. This result confirmed the production of the catecholate siderophore Bacillibactin, predicted by genome mining and reported as a putative secondary metabolite for this species [36,37]. Bacillibactin has been associated with the biocontrol activity of diverse beneficial strains [38,39] not only through iron scavenging but also by direct antimicrobial activity [39]. However, strain TSO55 exhibited low antifungal activity against *Alternaria alternata*, *Bipolaris sorokiniana*, and *Fusarium incarnatum*; this could be linked to the limited biosynthesis of this siderophore observed in vitro and the lack of production of other putative antimicrobial compounds indicated by the AntiSMASH analysis. To our knowledge, the antifungal capacity of *B. paranthracis* has been scarcely studied; for instance, a recent report indicated positive antifungal capacity against the phytopathogen *Macrophomina phaseolina*, but no details on the fungal growth inhibition percentage were given [40]. In contrast, other isolates from the *Bacillus cereus* group have been extensively searched for their antifungal properties, such as *B. cereus* and *B. thuringiensis*, for which antifungal mechanisms reported include the production of bacteriocins (e.g., cerein, entomocin, morrocin), enzymes (e.g., chitinases), lipopeptides (e.g., kurstakins, surfactins, fengycins, cerexins), and siderophores (e.g., bacillibactin, petrobactin), among others [41,42].

Since *B. paranthracis* was only recently described [43], there is not much information about this species; however, it has been reported as an emergent human pathogen, due to its pathogenic traits, such as the putative production of enterotoxins and cell lysis agents (i.e., phospholipase C and cytolysin), as well as motility, biofilm formation capacity, cytotoxicity, and antibiotic resistance [44]. In this regard, during the genomic analysis of strain TSO55, three CDSs of pathogenicity islands were detected, corresponding to two phospholipase C and one thiol-activated cytolysin, which are tissue-destructive exotoxins, thus emphasizing the harmful potential of the strain [36]. Furthermore, according to the analysis performed on PathogenFinder, *B. paranthracis* TSO55 is predicted as a human pathogen, due to its association with 52 families of known pathogens, such as *Bacillus cereus*, *B. anthracis*, and *B. thuringiensis*. In vitro growth of strain TSO55 on blood agar indicated that this strain causes beta hemolysis, thus reinforcing its pathogenic potential.

Another important aspect of the RAST prediction of the *B. paranthracis* TSO55 genome is that it showed a high prevalence of genes related to virulence, disease, and defense, as well as to resistance to antibiotics and toxic compounds. It has been documented that *B. paranthracis* has extensive genetic machinery for antibiotic resistance, with 39 antimicrobial resistance genes to 18 antibiotic classes [34]. Some reports indicate the presence of beta-lactamases and fosfomycin resistance as core genes for *B. paranthracis* [37], both of which were present in the genome of strain TSO55, along with genes for the resistance to vancomycin and tetracycline. While diverse mechanisms and genetic factors are required for exhibiting effective resistance [45], the prevalence of antimicrobial resistance genes in the strain TSO55 genome increases its latent risk, as its management with common antibiotics could become challenging. Furthermore, horizontal transfer of AMR genes among *B. cereus* group species is highly frequent [34], which also represents a risk for established bacteria to develop AMR if strain TSO55 is introduced in the soil microbiome [46].

Results of the in vitro evaluation of antibiotic susceptibility of strain TSO55 to beta-lactams and fluoroquinolones predicted by genome annotation indicated resistance to antibiotics clavulanic acid + amoxicillin and dicloxacillin, from the beta-lactams group, up to a concentration of 20 µg/mL (highest concentration evaluated). Conversely, the antibiotic ciprofloxacin from the fluoroquinolone group generated an inhibition halo which expanded by more than twice (from 0.688 to 1.6945) as the antibiotic concentration increased from 2 µg/mL to 20 µg/mL. Susceptibility was also detected to the antibiotics erythromycin and clarithromycin with a minimal inhibitory concentration of 2 µg/mL. The susceptibility of *B. paranthracis* to ciprofloxacin and erythromycin has been previously reported at concentrations of 5 and 15 µg, respectively [47]. The fact that any pathogenic strain present in the field exhibits broad antibiotic resistance is highly concerning for food security as, if the bacteria are transmitted to the grains, it could remain a latent risk despite food manufacturing processes [11,12].

Finally, when evaluating the impact of strain TSO55 inoculated on wheat seedlings, a significant decrease in wheat biometric parameters was noted, specifically a 4% decrease in total length and a 15% decrease in dry biomass. Thus, the inoculation of strain TSO55 did not promote plant growth, contrasting with previously published results [40]. A recent study reported a significant decrease in cotton plants’ dry weight after their inoculation with two *Bacillus* strains: *Bacillus coagulans* and *B. cereus*. However, only *B. coagulans* exhibited pathogenic potential, as it increased the incidence of damping-off [48]. In our study, despite hindering wheat plants’ development, no disease symptoms were detected, which suggests that growth regulators are involved during the infection process, instead of tissue-degrading enzymes [48].

Despite their hazardous potential, known pathogenic microbial species are often employed in plant growth promotion studies, without disclosing the inherent risk they represent to food safety. In this case, strain *B. paranthracis* TSO55 represents a health hazard for consumers, and its presence in water sources has already been correlated to major disease outbreaks [49,50]. Interestingly, despite its pathogenic potential, *B. paranthracis* has recently been explored as a promising probiotic [47,51]. Moreover, it has also been reported as a plant growth promoter, due to its high tolerance to osmotic and drought stresses [40]. However, this resistance could increase its potential risk, as it could remain viable through drying and manufacturing processes and thus affect consumers’ health. In addition, the introduction of human pathogenic strains on crop fields could facilitate their transfer to other crops and diverse sectors, such as houses, hospitals, and restaurants, through dispersion by wind (bioaerosols), soil erosion, and water runoff or leaching [9]. Thus, pathogenic bacteria presence in organic amendments represent a major emergent risk, due to direct human exposure and the transmission of antibiotic resistance genes and virulence-related factors [5,52]. For this reason, it is essential to implement preventive measures to treat and analyze organic amendments before land dispersal, to avoid the proliferation of potentially harmful strains, such as *Escherichia coli*, *Salmonella enterica*, and *Listeria monocytogenes* in agricultural soils. Furthermore, good agricultural practices, constant surveillance of the wheat-based product manufacturing chain, and implementation of safety measures could contribute to the mitigation of pathogenic contamination risk [11].

Overall, this study case presents an example of a pathogenic strain colonizing an agricultural field fertilized by organic amendments, while its absence in the field under conventional fertilization suggests it was introduced by the added organic matter. Our results highlight the importance of analyzing the microbial composition of organic fertilizers before application in the field, to limit the proliferation of potential pathogens. In the future, an analysis of the impact on the native microbial communities, following the application of organic matter, would be highly valuable to successfully and safely implement this agronomic practice.

## 4. Materials and Methods

### 4.1. Bacterial Isolation and Morphological Characterization

Bacterial strain TSO55 was isolated from soil in a wheat commercial field in the Yaqui Valley, Mexico (longitude 109°30′ to 110°37′ E; latitude 26°45′ to 27°33′ N) in 2014. The soil from which the isolation was performed was fertilized, for 10 years, with organic amendments (chicken manure, earthworm humus, and fish concentrate) to meet the nutrient requirements of wheat [3]. Bacterial isolation was carried out as described in [53]; in short, 10 g of the collected soil was placed in Erlenmeyer flasks containing 90 mL of sterile distilled water (121 °C and 15 psi for 15 min) and homogenized at 150 rpm for 1 h. Then, serial dilutions (1:10) up to 10^−6^ were carried out, and 100 µL was inoculated on Petri dishes containing nutrient agar (NA) as a culture medium, supplemented with 80 ppm of terbinafine, and incubated for 2 days at 28 °C. After this time, bacterial colonies were counted, picked, and purified by sequential streaking on NA. Of note, strain TSO55 was present in the soil of the study site (under organic amendments, as described previously) and was absent in soil under conventional (intensive) synthetic (urea, 250 Kg N ha^−1^; monoammonium phosphate, 100 Kg P ha^−1^) fertilization for 10 years [3]. Following isolation, strain TSO55 was grown on Petri dishes containing NA as a culture medium and cultivated for 24 h at 28 °C for its morphological characterization (colony border, shape, and color). Then, for microscopical characterization, a single colony was dispersed on 20 µL of sterile distilled water and fixed by heat to a glass slide for Gram staining, which was conducted using a Gram stain kit (HYCEL, Zapopan, Jalisco, México), following the manufacturer’s instructions. Finally, strain TSO55 was cryopreserved at −80 °C using nutrient broth (NB) as a culture medium and glycerol (30%), and stored at the Colección de Microorganismos Edáficos y Endófitos Nativos (COLMENA) [54,55].

### 4.2. Genomic Analysis

High-quality genomic DNA was extracted from a fresh culture of strain TSO55 cultivated for 48 h at 150 rpm and 30 °C on NB. To achieve this, 100 µL [1 × 10^7^ colony-forming units (CFU)/mL] of the cell suspension was lysed with 120 μL of TE buffer (pH 8; 10 mM Tris-HCl, 1 mM EDTA•Na_2_.) containing lysozyme (final concentration 0.1 mg/mL) and RNase A (final concentration 0.1 mg/mL), and incubated for 25 min at 37 °C. Then, proteinase K (final concentration 0.1 mg/mL) and sodium dodecyl sulfate (SDS; final concentration 0.5% *v*/*v*) were added and incubated for 5 min at 65 °C. Genomic DNA was purified using an equal volume of SPRI beads and resuspended in EB buffer (pH 8.0; 10 mM Tris-HCl). The total extracted DNA (80 ng/µL, 30 µL) was quantified with the Quant-iT dsDNA HS kit (ThermoFisher Scientific, Waltham, MA, USA) assay in a plate reader and diluted as needed [56].

DNA sequencing was performed on the Illumina NovaSeq 6000 (Illumina, San Diego, CA, USA) platform (2 × 250 bp). Library preparation was conducted using the Nextera XT Library Prep Kit (Illumina, San Diego, CA, USA), according to the manufacturer’s protocol, with the following modifications: the input DNA was doubled and PCR elongation time was increased to 45 s. DNA quantification and library preparation were carried out on a Hamilton Microlab STAR automated liquid handling system (Hamilton Bonaduz AG, Reno, NV, USA).

The genomic analysis was performed following the workflow reported by [57] and [56]. Briefly, Trimmomatic version 0.30 [58] was used to remove adapter sequences and eliminate low-quality bases. The SPAdes version 3.13.1 [59,60] generated a de novo assembly, and the contigs were ordered and compared with the genome of *Bacillus paranthracis* Mn5 ^T^ (GenBank accession number GCA_001883995.1), using Mauve Contig Mover 20150226 version build 10 [61,62]. Plasmid detection was performed with the online platform PlasmidFinder version 2.1 [63,64]. The genome sequence of strain TSO55 was analyzed for contamination using CheckM version 1.0.18 [65] and Quast version 4.4 [66], which was negative. To affiliate strain TSO55 at the species level, its genome was compared to the genomes of its more closely related strains (16S rRNA similarity ≥ 98.7%) by using the overall genome relatedness indices (OGRIs): average nucleotide identity (ANI) by the OrthoANI algorithm [67] and the Genome to Genome Distance Calculator (GGDC) version 3.0 by BLAST [68,69]. To confirm the taxonomic affiliation of strain TSO55, phylogenomic relationships were established using the genome sequences of the *Bacillus*-type strains closely related to strain TSO55. Thus, a phylogenomic tree was constructed using Type (Strain) Genome Server (TYGS), a high-throughput web server for genome-based prokaryote taxonomy [69,70,71]. The tree was inferred with FastME 2.1.6.1 [30] from Genome blast Distance Phylogeny method (GBDP) distances calculated from genome sequences.

### 4.3. Genome Annotation and Genome Mining

The genome annotation for strain TSO55 was performed using the Rapid Annotation using Subsystem Technology (RAST) server version 2.0 and the RASTtk pipeline based on the PathoSystems Resource Integration Center (PATRIC) [72,73,74]. A second annotation was carried out using Proksee [75,76], which incorporates the Rapid Prokaryotic Genome Annotation (Prokka Software version 1.14.6, Tool version ‘Proksee’ 1.2.0) [77], resulting in the generation of the circular chromosome map of strain TSO55. This map includes coding sequences (CDSs), tRNAs, rRNAs, guanine-cytosine (GC), and skew content. Additionally, the Comprehensive Antibiotic Resistance Database (CARD Software RGI 6.0.3) tool [78] was used to identify resistance genes within the genome, which were added to the map. The protein sequence FASTA file annotated using RAST was uploaded to the prediction tool PGPT-Pred in PlaBAse [79,80], a prediction tool for bacterial plant growth-promoting traits. Likewise, to identify biosynthetic gene clusters (BGCs) associated with biocontrol, the genome of strain TSO55 was submitted to the Antibiotics & Secondary Metabolite Analysis Shell (AntiSMASH) 7.1 web server [81,82], under the ‘relaxed’ parameter. Additionally, to predict human pathogenicity of strain TSO55, the PathogenFinder 1.1 online platform was used [83,84], both tools available on the website of the Center for Genomic Epidemiology (https://genomicepidemiology.org/services/, accessed on 15 January 2025).

### 4.4. In Vitro Evaluation of Predicted Traits of Strain TSO55: Plant Growth Promotion, Biocontrol, Antibiotic Susceptibility, and Pathogenicity

For in vitro evaluation of genome-mining traits, firstly, an overnight culture (24 h) of strain TSO55 was prepared by inoculating 1 × 10^4^ CFU on nutrient broth (NB) as a culture medium and agitating at 120 rpm for 24 h. Then, bacterial cells were centrifuged for 5 min to 5000 rpm, and they were washed twice with sterile distilled water and adjusted to a 630 nm absorbance of 0.5 (1 × 10^6^ CFU/mL). Then, strain TSO55 (10 µL) was inoculated on four specific media to evaluate diverse biochemical traits related to beneficial traits for plants, such as siderophore production (CAS medium), phosphate solubilization (PVK medium), indole production (colorimetric quantification using Salkowsky reagent), and potassium solubilization (Aleksandrow agar), according to previously published protocols [3,85,86,87], which was carried out in triplicate.

For biocontrol assays, strain TSO55 inoculum was generated as previously mentioned. Furthermore, three fungal species belonging to the COLMENA collection and isolated from wheat leaf lesions were used, namely *Bipolaris sorokiniana* TPQ3, *Fusarium incarnatum* TF14, and *Alternaria alternata* TF17 [54]. These fungal species have been reported as phytopathogens affecting roots, stems, and leaves of diverse agricultural and horticultural crops besides wheat [88,89,90,91]. Thus, for fungal spore production, Petri dishes containing potato dextrose agar (PDA) as a culture medium were inoculated with 1 × 10^4^ CFU, using fresh fungal mycelium, and incubated at 30 °C for 7 days. Then, fungal spores were collected by adding 10 mL of sterile distilled water with 0.01% Tween 80 [92]. The spores were scraped from the surface of the fungal colony and enumerated in a Neubauer chamber. Fungal spore suspension was then adjusted to 1 × 10^6^ spores/mL. Dual confrontation assays were performed according to the protocol described by [92] with modifications. Briefly, 10 µL of fungal spore suspension (1 × 10^6^ spores/mL) was inoculated at the center of Petri dishes containing PDA. Then, 10 µL (1 × 10^6^ CFU) of strain TSO55 was inoculated on each side of these Petri dishes, at 1 cm from the border. For the control of fungal growth, bacteria were not inoculated. Assays were performed in triplicate. Inoculated plates were incubated at 25 °C for 7 days. Fungal growth inhibition was measured after 7 days through image analysis with ImageJ software (version 1.53) [93]. The control growth area was established as 100%, and the percentage of fungal inhibition caused by the bacterial colonies was determined.

Furthermore, antibiotic susceptibility was evaluated by the disk diffusion method [47] on nutrient agar (NA) plates and by microdilution on microplates containing nutrient broth. For this, 100 µL of bacterial suspension of strain TSO55, prepared as described previously, was inoculated on the surface of the plate or in the well, respectively. For the disk diffusion method, filter squares (0.5 cm) were placed on the surface of the inoculated plates, and 10 µL of aqueous solutions of six commercial antibiotics, or antibiotic combinations (ciprofloxacin; erythromycin; dicloxacillin; sulfamethoxazole and trimethoprim; clarithromycin; and clavulanic acid and amoxicillin) at concentrations of 0.02, 0.2, 2, and 20 µg/mL, were added to each filter. For the microplate assay, antibiotics were added to the wells to final concentrations of 0.02, 0.2, 2.0, and 20.0 µg/mL, and bacterial growth was compared to a control without antibiotic addition. Inoculated plates were incubated for 24 h at 28 °C, while microplates were under the same conditions plus agitation at 110 rpm. Susceptibility was determined by identifying the minimal inhibitory concentration (MIC), both on plates and microplates, and by measuring the diameter of the inhibition halo of the bacteria in the disk diffusion assay.

Finally, the pathogenic potential of strain TSO55 was evaluated through a hemolysis assay, by inoculating 10 µL of bacterial spore suspension on a blood agar medium. The hemolytic reaction was indicated by the formation of a halo around the colony and its characteristics defined the hemolysis type [94].

### 4.5. In Vivo Impact of Strain TSO55 on Wheat Growth

Durum wheat seeds (CIRNO C2008) were sowed on a 128-cell seedling tray filled with a commercial growing medium (PROMIX^®^FLX, Premier Horticulture Inc., Delson, QC, Canada) and inoculated directly in the soil in the cell with 1 mL of bacterial inoculum described previously (1 × 10^6^ CFU/mL). For the control, 1 mL of distilled sterile water was added to each cell. Seedlings were kept for 10 days at 25 °C and watered uniformly every other day. The average daytime and nighttime temperatures were 23 °C and 30 °C, respectively, and the average relative humidity was 35%. At the end of the cultivation period, seedlings were extracted from the tray to measure biometric parameters, such as total length, stem length, and root length. After a 3-day drying period at around 35 °C, additional biometric parameters were measured for each plant, such as total dry weight, stem dry weight, and root dry weight [85,95]. Data were analyzed using Rstudio software (version 2024.04.2+764). The normal distribution of data was verified by the Shapiro–Wilk normality test. Statistical analysis of non-parametric data was conducted using the Kruskal–Wallis ANOVA on ranks. When significant differences were found (*p*-value ≤ 0.05), pairwise comparisons were performed using the post hoc Wilcoxon method with a 95% confidence interval.

## 5. Conclusions

Organic amendments are an effective way to increase the organic matter and fertility of soils, but due to some of their origins (i.e., manure, decomposing waste), could also represent a risk of introducing potentially dangerous microorganisms to the soil. In this sense, we analyzed a bacterial strain isolated from a wheat field under organic supplementation practices. After a thorough examination of the genetic traits of strain TSO55, the results indicated that this strain was identified as a human pathogen, *Bacillus paranthracis*, which also possessed several antibiotic resistance genes, making it a potential risk to food safety. Moreover, according to our results, *B. paranthracis* TSO55 did not possess any agricultural application potential, neither as a biofertilizer nor as a biopesticide. The study and results presented here are an example of the potential risks that can be encountered when introducing organic amendments of unknown origin or without previous microbiological analyses. We suggest that industrial organic amendments should be treated to decrease their pathogenic load and microbiologically analyzed before being sold for use in agricultural fields, to ensure the safety of crops and consumers. Moreover, our findings emphasize the importance of conducting extensive and deeper genomic and functional analyses to obtain a precise taxonomic affiliation of any introduced microbial inoculant, as this information allows us to elucidate the beneficial bioactivity and/or harmful potential of the introduced microbiome. Overall, these types of analyses represent a robust sorting tool that could accelerate the approval or refusal of microbial inoculants, guaranteeing their quality and safety according to national legal regulations [96], contributing in this way to more sustainable agricultural practices that promote One Health worldwide.

## Figures and Tables

**Table 1:** 16S rRNA gene and OGRI-based taxonomic affiliation of strain TSO55.

Taxon Name	Strain	GenBank Accession Number	16S Similarity (%)	Strain	ANIb	ANIm	OrthoANI	GGDC (Formula 2) (%)
*Bacillus paranthracis*	Mn5 ^T^	MACE01000012	100	Mn5 ^T^	97.4	97.81	97.72	79.3
*Bacillus nitratireducens*	4049 ^T^	KJ812430	100	4049 ^T^	89.51	90.43	90	39.7
*Bacillus tropicus*	N24 ^T^	MACG01000025	100	N24 ^T^	95	95.61	95.45	62.9
*Bacillus anthracis*	Ames	AE016879	100	Vollum ^T^	94.71	95.27	95.07	60.8
*Bacillus cereus*	ATCC 14579 ^T^	AE016877	99.92	ATCC 14579 ^T^	91.35	92.03	91.78	45.4
*Bacillus albus*	N35-10-2 ^T^	MAOE01000087	99.92	N35-10-2 ^T^	92.8	93.7	93.3	52.3
*Bacillus paramycoides*	NH24A2 ^T^	MAOI01000012	99.92	NH24A2 ^T^	88.35	89.47	88.84	36.8
*Bacillus luti*	TD41 ^T^	MACI01000041	99.92	TD41 ^T^	90.96	91.8	91.59	44.4
*Bacillus mobilis*	0711P9-1 ^T^	MACF01000036	99.92	0711P9-1 ^T^	92.67	93.22	93.07	50.2
*Bacillus sanguinis*	BML-BC004 ^T^	MW674727	99.92	BML-BC004 ^T^	93.37	94.01	93.83	54.1
*Bacillus paramobilis*	BML-BC017 ^T^	MW674728	99.92	BML-BC017 ^T^	92.91	93.48	93.33	58.29
*Bacillus toyonensis*	BCT-7112 ^T^	CP006863	99.84	BCT-7112 ^T^	90.34	91.28	90.94	42.8
*Bacillus wiedmannii*	FSL W8-0169 ^T^	LOBC01000053	99.84	FSL W8-0169 ^T^	92.69	93.41	93.28	51.2
*Bacillus proteolyticus*	TD42 ^T^	MACH01000033	99.84	TD42 ^T^	89.33	90.3	89.67	39.4
*Bacillus pacificus*	EB422 ^T^	KJ812450	99.84	EB422 ^T^	95.45	96.09	95.93	66.2
*Bacillus fungorum*	17-SMS-01 ^T^	MG601116	99.76	17-SMS-01 ^T^	92.84	93.91	93.53	53.5
*Bacillus arachidis*	SY8 ^T^	OM062591	99.76	SY8 ^T^	81.35	85.99	82.21	26.7
*Bacillus pseudomycoides*	DSM 12442 ^T^	ACMX01000133	99.69	DSM 12442 ^T^	81.48	86.03	82.23	26.8
*Bacillus hominis*	BML-BC059 ^T^	MW674729	99.69	BML-BC059 ^T^	89.01	90.14	89.58	39
*Bacillus mycoides*	DSM 2048 ^T^	ACMU01000002	99.53	DSM 2048 ^T^	88.82	89.89	89.44	38.2
*Bacillus gaemokensis*	KCTC 13318 ^T^	LTAQ01000012	99.05	KCTC 13318 ^T^	81.31	85.83	82.07	26.5
*Bacillus rhizoplanae*	JJ-63 ^T^	OM391995	98.9	JJ-63 ^T^	77.71	85.02	78.67	23.9

^T^ = Type strain.

**Table 2:** In vitro evaluation of antibiotic susceptibility of strain TSO55 grown on nutrient agar medium by the disk diffusion method.

		Inhibition Halo Diameter (cm)			
	Concentration	0.02 µg/mL	0.2 µg/mL	2 µg/mL	20 µg/mL
Antibiotic					
Ciprofloxacin		NI	NI	0.688	1.6945
Erythromycin		NI	NI	0.818	1.928
Dicloxacillin		NI	NI	NI	NI
Sulfamethoxazole and trimethoprim		NI	NI	NI	NI
Clarithromycin		NI	NI	1.121	2.487
Clavulanic acid and amoxicillin		NI	NI	NI	NI

NI: No inhibition.

**Table 3:** Biometric parameters of wheat seedlings without (control) and with the inoculation of strain TSO55 (TSO55 treatment) measured after 10 days of growth.

		Control	TSO55 Treatment	Reduction, %
Length (cm)	Stem	18.4 ± 2.1 ^a^	18.3 ± 2.2 ^a^	0.4
	Roots	8.6 ± 1.5 ^a^	7.9 ± 1.6 ^b^	8.4
	Total	27.1 ± 2.6 ^a^	26.0 ± 3.0 ^b^	4.2
Dry weight (mg)	Stem	18.9 ± 4.8 ^a^	16.8 ± 3.7 ^b^	11.1
	Roots	29.5 ± 8.4 ^a^	24.0 ± 7.9 ^b^	18.6
	Total	0.0488 ± 0.0099 ^a^	0.0414 ± 0.0095 ^b^	15.16

The presented data are results and standard errors from 128 replicates. Results with different letters are significantly different according to the Kruskal–Wallis test and Wilcoxon post hoc treatment (*p*-value ≤ 0.05).

## Data Availability

The complete genome sequence of strain TSO55 was deposited in DDBJ/ENA/GenBank and is openly available in NCBI under accession number JBIQNN000000000, BioProject number PRJNA1080047, and BioSample number SAMN44426338.
